# Physicians' prediction for the assessment of atypical pathogens in respiratory tract infections

**DOI:** 10.1002/jgf2.350

**Published:** 2020-06-25

**Authors:** Satoshi Suzuki, Naoto Ishimaru, Yusaku Akashi, Yuto Takeuchi, Atsuo Ueda, Akihito Ushiki, Saori Kinami, Hiromichi Suzuki, Yasuharu Tokuda, Tetsuhiro Maeno

**Affiliations:** ^1^ Division of General Medicine Tone Chuo Hospital Gunma Japan; ^2^ Department of General Internal Medicine Akashi Medical Center Japan; ^3^ Division of Infectious Diseases Department of Medicine Tsukuba Medical Center Hospital Tsukuba Ibaraki Japan; ^4^ Department of Clinical Laboratory Tsukuba Medical Center Hospital Tsukuba Japan; ^5^ Department of Clinical Laboratory Tone Chuo Hospital Gunma Japan; ^6^ Muribushi Okinawa for Teaching Hospitals Okinawa Japan; ^7^ Faculty of Medicine University of Tsukuba Tsukuba Japan

**Keywords:** Atypical pathogen, *Bordetella pertussis*, *Chlamydophila pneumoniae*, Infectious diseases, *Mycoplasma pneumoniae*, the Japanese Respiratory Society guideline

## Abstract

**Background:**

Patients with acute respiratory tract infections are frequently prescribed antimicrobials despite high rates of virus detection. Physicians may overprescribe antimicrobials owing to the concern of bacterial infections, including those because of atypical pathogens. We investigated the accuracy of clinical predictions concerning atypical pathogen infections.

**Methods:**

We prospectively enrolled adult patients who presented with a fever and cough in outpatient clinics between December 2016 and August 2018. After taking a history and performing physical examinations, physicians predicted the possibility of respiratory infections because of atypical pathogens. Disease probabilities were categorized into 3 grades (high: ≥50%, intermediate: 20% ≥ and <50%, and low: <20%) and were judged by physicians who were taking care of the patients. Confirmation of atypical pathogens was performed by comprehensive molecular analyses of respiratory samples.

**Results:**

Atypical pathogens were detected in 21 of 210 patients. A close contact history (odds ratio [OR]: 11.4, 95% confidence interval [CI]: 2.4‐53.5) and the presence of pneumonia (OR: 12.9, CI: 4.3‐39.2) were associated with the detections. Atypical pathogens were detected in 32.3% of high‐probability cases (10/31), while atypical pathogens were only detected in 8.8% of intermediate‐probability cases (8/91) and 3.4% of low‐probability cases (3/88) (*P* < .001).

**Conclusions:**

The current study indicates that physicians’ predictions were associated with the detection of atypical pathogens; however, overestimation was observed.

## Graphical abstract

Our study indicates that while physicians' predictions were associated with the detection of atypical pathogens, overestimation was observed. The criteria for atypical pneumonia did not perform well for accurately diagnosing atypical pathogen infection in patients with a fever and cough.

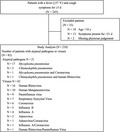

## 1 INTRODUCTION

Overprescription of antimicrobials has contributed to the increased prevalence of antimicrobial‐resistant bacteria.^1^, ^2^ To counteract this trend, national action plans have been developed in many countries.^3^ The majority of antimicrobial agents, reportedly up to 90%, are prescribed in an outpatient setting.^4^ Acute respiratory tract infections are the primary reason for these prescriptions,^5^ and antimicrobial agents are prescribed in up to 70% of patients with upper respiratory tract infections or acute bronchitis in outpatients care settings in both Japan ^6^ and the United States,^7^ despite high virus detection rates in these cases.^8^, ^9^

*Mycoplasma pneumoniae*, *Chlamydophila pneumonia*, and *Bordetella pertussis* have been recognized as the main causes of bacterial bronchitis.^10^ Differentiation between bacteria and viruses is considered to be challenging, and a previous study reported that there were no specific signs or symptoms associated with *M pneumoniae* infections.^11^ Concerns regarding possible bacterial infections may lead physicians to prescribe antimicrobials, so detecting clues to accurately predict these infections may promote antimicrobial stewardship. Recently, diagnostic scoring criteria for considering atypical pathogen infections among adult pneumonia patients were published by the Japanese Respiratory Society (JRS) and are now widely used in Japan.^12^ These criteria were developed to support the diagnosis of atypical pathogens among pneumonia patients, but the criteria were not applied to other respiratory infections.

In this study, we investigated the epidemiology and characteristics of atypical pathogen infections in the outpatient clinic setting using comprehensive molecular analyses in order to evaluate physicians’ diagnostic predictions and the performance of the JRS criteria for diagnosing atypical pathogen infections.

## 2 MATERIALS AND METHODS

This study was a prospective observational design and performed at the ambulatory clinics of two acute care hospitals between December 2016 and August 2018 in Japan. Written informed consent was obtained from all participants in this study. Ethical approval was granted by the Review Board Committee of each hospital.

**Figure 1:**
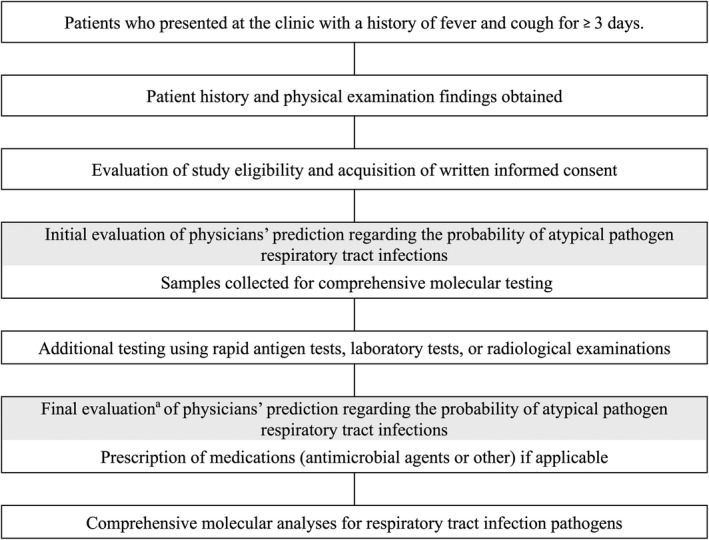
Flowchart of the study process. When additional tests were not ordered by physicians during care for patients, the prediction of the initial evaluation was used as the final prediction for atypical pathogen infections

All patients who met the study inclusion criteria had respiratory samples obtained for a comprehensive molecular examination (Figure 1). Physicians initially predicted the potential for atypical pathogen infections after obtaining the patient history and conducting a physical examination. Each physician documented their judgment concerning the potential for atypical pathogen infections as 1 of 3 grades (high: ≥50%, intermediate: 20% ≥ and <50%, and low: <20%). All of the judgments were performed subjectively by the physicians taking care of the patients. If trainees examined study patients, all predictions were performed under the instruction of the attending physicians.

If further tests, including antigen testing (influenza antigen testing, pneumococcal urinary antigen testing, legionella urinary antigen testing, *Mycoplasma pneumoniae* antigen testing, and rapid antigen detection test for *group A streptococcus*), blood tests, or radiological imaging, were performed, the diagnostic predictions were reevaluated prior to the patient leaving the clinic on the same day. A laboratory test for *M pneumoniae* using the particle agglutination (PA) antibody was not available at the study hospitals in a single day. The reevaluation of judgments for the potential for atypical pathogen infections was documented by the same grading system as mentioned above. Each reevaluation was performed by each physician who was taking care of the patients without instruction from other physicians, except for trainees; however, strict rules prohibiting talking about patients between the first and second evaluations were not implemented.

Comprehensive molecular examinations were performed at a later date, and the results were made not available to physicians during the evaluation.

### 2.1 Inclusion and exclusion criteria for patients

All patients who presented at the clinic with both a fever (1 degree higher than their baseline body temperature or a body temperature >37°C) and cough for at least 3 days were enrolled in this study. Pediatric patients (age <18 years), patients with unstable physical conditions (eg, shock), a history of multiple exacerbations of chronic pulmonary disease, an apparent history or presence of dysphagia, presence of obstructive pneumonia, lung abscess, empyema, healthcare‐associated pneumonia or hospital‐onset pneumonia referred from other facilities, tuberculosis, nontuberculous mycobacterium lung infections, pneumomycosis, sinusitis, or tonsillitis were excluded from this study. In addition, patients with a history of a fever or cough for more than 21 days or patients without documentation of their physicians’ prediction regarding the probability of atypical pathogen infections were also excluded.

### 2.2 Data collection and physicians' prediction regarding the probability of detecting atypical pathogens

As background data, we collected information on the age, gender, visiting month, comorbidities, close contact with patients confirmed to have atypical pathogen infections, history of preceding antimicrobial use, history of signs and symptoms (rhinorrhea, sputum, severe cough, sore throat, myalgia, arthralgia, diarrhea, and rash), duration of symptoms at the time of clinical visits, findings of chest auscultation, laboratory findings (white blood cell [WBC] count and C‐reactive protein [CRP] levels), and presence of pneumonia. Severe cough was defined as cough with vomiting, sleep disturbance, or persistent cough. Pneumonia was diagnosed based on clinical symptoms, signs, and radiological findings compatible with pneumonia, without other causes attributed to abnormal radiological findings. All images were reviewed by a board‐certified pulmonary physician for the determination of the final diagnosis.

We calculated the scores for atypical pathogens by the published JRS guidelines.^12^ Scores were determined by the following items: (a) age <60 years; (b) no or only minor underlying diseases; (c) persistent cough; (d) scant chest auscultatory findings; (e) no sputum or no identified etiological agent by rapid diagnosis; and (f) white blood cell count <10 000/μL. The scoring criteria without laboratory tests consisted of items (a) through (e), and a score ≥3 was considered indicative of an atypical pathogen pneumonia. The scoring criteria with laboratory tests consisted of items (a) to (f), and a score ≥4 was considered indicative of an atypical pathogen pneumonia.

### 2.3 A comprehensive molecular analysis for atypical pathogens and viruses

Nasopharyngeal and oropharyngeal samples were obtained from all study participants at the time of the initial evaluation. The samples were stored at −80°C until they were used for analyses unless molecular analyses were performed immediately after samples were obtained. Molecular analyses were performed with the FilmArray® system and the FilmArray® Respiratory Panel tests for a comprehensive panel of 20 respiratory viruses and bacteria.^13^ Additional molecular analyses for *M pneumoniae* were performed using oropharyngeal samples and the GENECUBE® system^14^ because the FilmArray® system used nasopharyngeal samples, which have a lower *M pneumoniae* detection rate than oropharyngeal samples.^15^

### 2.4 Statistical analyses

Categorical and continuous variables were compared using Fisher's exact test and the Mann‐Whitney U‐test, where appropriate. A multivariable logistic regression model was constructed to identify variables significantly associated with atypical pathogen positivity. Variables with *P*‐value <.05 in the univariate analyses were included in the multivariable model. A *P*‐value less than .05 was considered significant. All statistical analyses were performed with the SPSS version 20 software program (IBM).

## 3 RESULTS

### 3.1 Study case selection and detected pathogens

**Figure 2:**
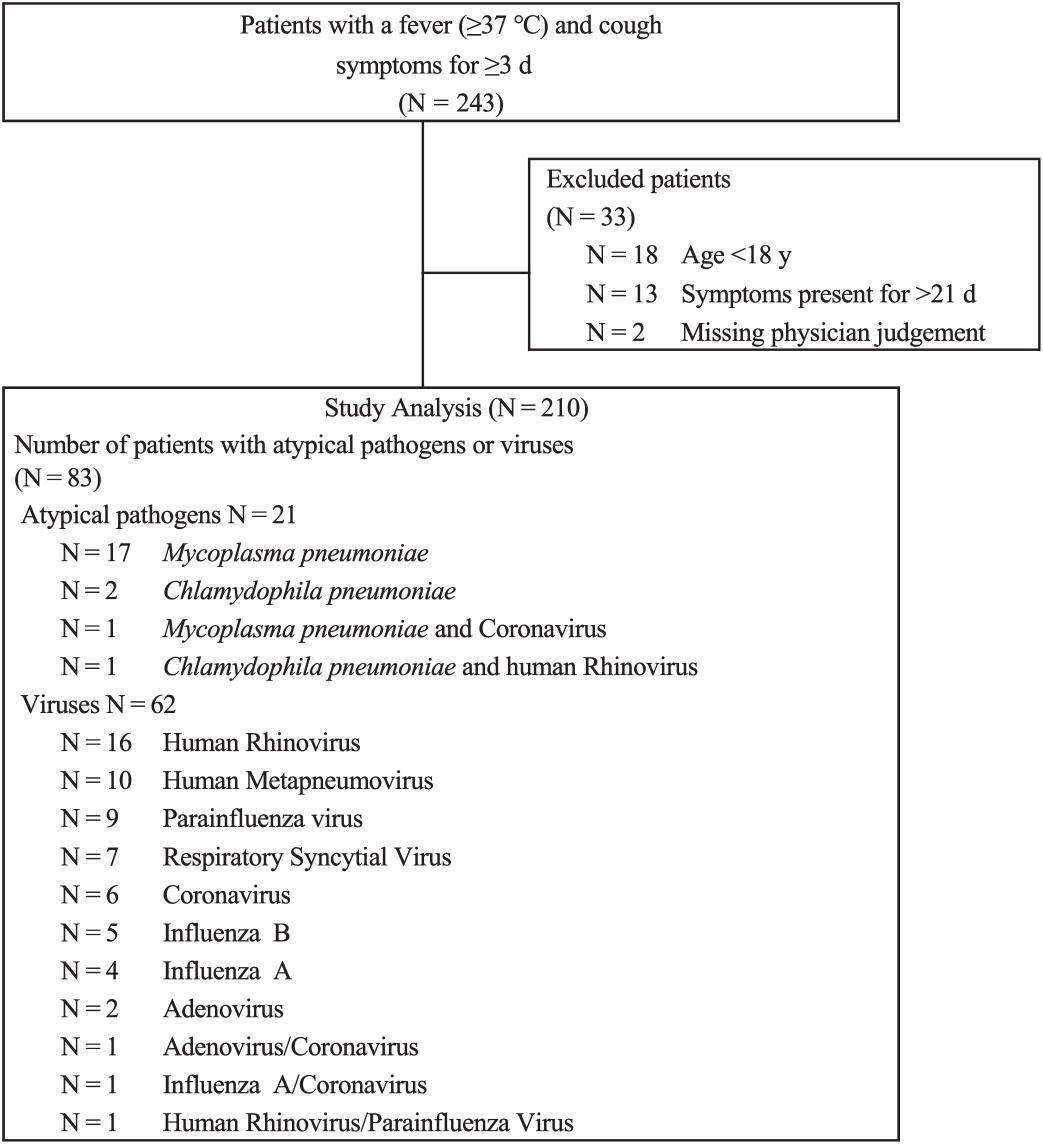
Flowchart describing patient enrollment, case selection, and pathogens detected in this study

The flowchart describing the case selection process is shown in Figure 2. A total of 243 patients agreed to participate in the study, and their samples were examined using a comprehensive molecular analysis for atypical pathogens and viruses. Among the 243 patients, pediatric patients (n = 18), patients with chronic symptoms (n = 13), and patients without documentation of physicians’ predictions regarding atypical pathogen infections (n = 2) were excluded, leading to a final study population of 210 patients. Of the 210 patients, 18 (8.6%) were referred to participating hospitals, and pneumonia had been confirmed in 9 of these patients (4.3%) prior to their hospital arrival.

Atypical pathogens were detected in 21 of 210 study patients (10%, 18; *M pneumoniae*, 3; *C pneumoniae*). Among these 21 patients, two had both atypical pathogens and viruses (one case of *M pneumoniae* with coronavirus and one case of *C pneumoniae* with human rhinovirus). Of the 18 patients with *M pneumoniae,* the bacteria were detected by both FilmArray® and GENECUBE® systems in 13 patients, by only the FilmArray® system in one patient, and by only the GENECUBE® system in four patients. Viral infections without accompanying atypical pathogen infections were found in 62 patients (29.5%).

### 3.2 Clinical characteristics of patients with atypical pathogen respiratory tract infections

Table 1 presents the clinical characteristics of patients with atypical pathogen‐positive respiratory tract infections and the comparison between the characteristics of patients with atypical pathogen‐positive and pathogen‐negative respiratory tract infections. The median age of the 21 positive patients was 36.0 years (interquartile range [IQR]: 32.0‐44.0 years old, female: 61.9%). Comorbidities were described in one patient in the positive patient group. A history of close contact with other persons with atypical pathogen infections was noted in 23.8%, while a history of antimicrobial use was noted in 47.6% of positive patients. For clinical symptoms and signs, sputum or productive cough was the most frequent (90.5%), followed by sore throat (42.9%), myalgia or arthralgia (38.1%), rhinorrhea (14.3%), and diarrhea (9.5%). Pneumonia was the most common diagnosis, found in 15 patients (71.4%). Hospitalization on the day of the evaluation was required in 1 patient among the 21 positive cases. No patients died during the study period. A comparison between positive and negative patients showed that a history of close contact (adjusted odds ratio [OR]: 11.4, 95% confidence interval [CI]: 2.4 ‐53.5) and a final diagnosis of pneumonia (OR: 12.9, 95% CI: 4.3‐39.2) were significant factors associated with atypical pathogen–induced respiratory tract infection.

**Table 1:** A comparison of the clinical characteristics between patients with atypical pathogen‐positive respiratory tract infections and those with atypical pathogen‐negative respiratory tract infections

	Atypical pathogen^a^‐positive	Atypical pathogen‐negative	Crude *P*‐value	Adjusted *P*‐value	Odds ratio (95% CI)
n	21	189			
Age (y)	36 [32, 44]	39 [28, 60]	.37		
Female	13 (61.9)	113 (59.8)	.99		
Comorbidities	1 (4.8)	26 (13.8)	.49		
Asthma	0 (0)	4 (2.1)	.99		
Immunosuppressive state	0 (0)	3 (1.6)	.99		
Season (August‐December)	11 (52.4)	73 (38.6)	.25		
Close contact	5 (23.8)	6 (3.2)	<.01	<.01	11.37 (2.42‐53.46)
Preceding antimicrobial use	10 (47.6)	43 (22.8)	.02	.06	2.77 (0.97‐7.94)
Macrolides, quinolones, or tetracyclines	2 (9.5)	16 (8.5)	.70		
Onset to evaluation (days)	7 [6, 10]	7 [5, 11]	.67		
Rhinorrhea	3 (14.3)	30 (15.9)	.99		
Sputum or productive cough	19 (90.5)	147 (77.8)	.26		
Severe cough	12 (57.1)	93 (49.2)	.65		
Sore throat	9 (42.9)	108 (57.1)	.25		
Myalgia or arthralgia	8 (38.1)	62 (32.8)	.63		
Diarrhea	2 (9.5)	14 (7.4)	.67		
Crackles on auscultation	1 (4.8)	22 (11.6)	.48		
Skin rashes	0 (0.0)	9 (4.8)	.60		
WBC count (/μL)	8050 [7075, 9300]	7460 [5673, 9725]	.43		
CRP (mg/dL)	3.84 [2.52, 10.19]	3.32 [1.17, 6.63]	.29		
Diagnosis					
Pneumonia	15 (71.4)	32 (16.9)	<.01	<.01	12.91 (4.25‐39.18)
Bronchitis/URI/others^b^	6 (28.6)	157 (83.1)^b^			
Required hospitalization	1 (4.8)	21 (11.1)	.71		

Note

Categorical data are presented as numbers (proportion, %).

Continuous data are presented as medians with the interquartile range.

Abbreviations: CRP, C‐reactive protein; URI, upper respiratory infection; WBC, white blood cell

^a^ *Mycoplasma pneumoniae* (n = 19), *Chlamydophila pneumoniae* (n = 2).

^b^ Others (n = 4) include infectious mononucleosis (n = 3) and Japanese spotted fever (n = 1).

### 3.3 Physicians' predictions regarding the probability of atypical pathogen respiratory tract infections

As shown in Table 2, after obtaining patients' medical history and physical examination findings, 25 physicians assessed the probability of atypical pathogen respiratory tract infections (31 high, 91 intermediate, and 88 low). Of the 31 patients categorized as having a high probability of atypical pathogen infection, 16 were later diagnosed with pneumonia. Atypical pathogens were detected in 32.3% of high‐probability cases (10/31), while atypical pathogens were only detected in 3.4% of low‐probability cases (3/88, *P* < .001). Among pneumonia patients (n = 47), atypical pathogens were detected in half of high‐probability cases (8/16).

**Table 2:** Physicians’ predictions of the probability of atypical pathogen respiratory tract infections

Initial physician's prediction after taking a medical history and performing a physical examination			Final physician's prediction after additional testing^c^		
Probability^a^	Atypical pathogens detected	*P*‐value^b^	Probability^a^	Atypical pathogens detected	*p*‐value^b^
(a) All patients (n = 210)					
High (n = 31)	10 (32.3%)	<.001	High (n = 41)	13 (31.7%)	<.001
Intermediate (n = 91)	8 (8.8%)		Intermediate (n = 80)	3 (3.8%)	
Low (n = 88)	3 (3.4%)		Low (n = 89)	5 (5.6%)	
(b) Pneumonia patients (n = 47)					
High (n = 16)	8 (50.0%)	.19	High (n = 23)	10 (43.5%)	.07
Intermediate (n = 22)	5 (22.7%)		Intermediate (n = 13)	1 (7.7%)	
Low (n = 9)	2 (22.2%)		Low (n = 11)	4 (36.4%)	

^a^ Physician's prediction of the probability of atypical pathogen respiratory tract infections (≥50%: high, ≥20% and <50%: intermediate, and < 20%: low).

^b^ Comparison of the detection rates of atypical pathogens with the physician's prediction.

^c^ Additional tests were performed in 136 of 210 patients (64.8%): 42 [20.0%], rapid antigen testing; 98 [46.7%], blood examination; and 118 [56.2%], radiological examination).

Additional tests were performed in 136 of 210 patients (64.8%), with 42 (20.0%) undergoing rapid antigen testing, 98 (46.7%) undergoing blood tests, and 118 (56.2%) undergoing radiological examinations. Among the 42 patients undergoing rapid antigen testing, 10 received *Mycoplasma pneumoniae* antigen testing. Of these 10 patients, there were none with positive results on antigen testing. Following these, physicians changed their predictions for 29 patients (13.8%). Based on this revised prediction, the detection rate for atypical pathogens was 31.7% among high‐probability cases (13/41), 3.8% among moderate‐probability cases (3/80), and 5.6% among low‐probability cases (5/89), and these results were similar to those of the initial physician predictions.

### 3.4 Performance of atypical pathogen diagnostic scoring criteria based on the Japanese guideline

Using the diagnostic scoring criteria for atypical pathogens among pneumonia patients, 30 (63.8%; 30/47) met the score (≥3) for the criteria without laboratory tests, and 19 (45.2%; 19/42) met the score (≥4) for the criteria with laboratory tests. The sensitivity and specificity were 100% (95% CI: 69.8%‐100%) and 53.1% (95% CI: 34.7%‐70.9%), respectively, for the criteria without laboratory tests, and 100% (95% CI: 61.5%‐100%) and 74.2% (95% CI: 55.4%‐88.1%), respectively, for the criteria with laboratory tests (Table 3).

**Table 3:** Performance of the atypical pathogen diagnostic scoring criteria based on the Japanese guidelines^a^

Pneumonia patients					
	Criteria without laboratory tests^b^ (n = 47)			Criteria with laboratory tests^c^ (n = 42)	
	Atypical pathogens			Atypical pathogens	
	Positive	Negative		Positive	Negative
Score ≥ 3	15	15	Score ≥ 4	11	8
Score < 3	0	17	Score < 4	0	23
Total	15	32	Total	11	31
Sensitivity	100%	(69.8%‐100%)	Sensitivity	100%	(61.5%‐100%)
Specificity	53.1%	(34.7%‐70.9%)	Specificity	74.2%	(55.4%‐88.1%)
PPV	50%	(31.3%‐‐68.7%)	PPV	57.9%	(33.5%‐79.7%)
NPV	100%	(72.7%‐100%)	NPV	100%	(78.9%‐100%)

Note

The sensitivity, specificity, PPV, and NPV are provided with 95% confidence intervals.

Abbreviations: NPV, negative predictive value; PPV, positive predictive value.

^a^ The diagnostic scoring criteria for determining atypical pathogen infections were published by the Japanese Respiratory Society (12). The scores were based on the following factors: (a) age <60 y; (b) no or only minor underlying diseases; (c) persistent cough; (d) scant chest auscultatory findings; (e) no sputum or no identified etiological agent by a rapid diagnosis; and (f) WBC <10 000/μL.

^b^ The scoring criteria without laboratory testing consisted of factors (a) to (e), and a score ≥3 was considered indicative of atypical pathogen infection.

^c^ The scoring criteria with laboratory testing consisted of factors (a) to (f), and a score ≥ 4 was considered indicative of atypical pathogen infection.

## 4 DISCUSSION

Using comprehensive molecular analyses for respiratory pathogens, atypical pathogens were confirmed in approximately 10% of all patients with a fever and cough lasting more than 3 days. A history of close contact with other persons with atypical pathogen infection and the presence of pneumonia were factors significantly associated with the detection of atypical pathogens among these patients. While physicians’ predictions were associated with molecular detection rates of atypical pathogens, the overall detection rates were only about half of those predicted by physicians. Diagnostic scoring for atypical pathogen based on the Japanese guideline has high sensitivity and moderate specificity for detecting atypical pathogens in pneumonia patients.

Bacterial infections are generally reported to account for 5%‐10% of acute bronchitis cases.^16^ A recent multicenter European study examining the etiology of acute respiratory infection in an adult primary care setting reported that the detection rates of *M pneumoniae*, *B pertussis*, and *C pneumoniae* were 4.3%, 2.8%, and 5.0%, respectively, for community‐acquired pneumonia and 4.9%, 3.1%, and 5.3%, respectively, for lower respiratory tract symptoms without community‐acquired pneumonia.^17^ In the current study, the detection rates of *M pneumoniae* and *C pneumoniae* were 8.6% and 1.4%, respectively, which are similar to what has been previously reported.

The prediction of atypical pathogens among respiratory infections is challenging. *M pneumoniae* or *C pneumoniae* infections have shown a variety of symptoms, signs, and clinical presentations.^18^ Typical findings for pertussis infections, including whooping cough and marked lymphocytosis,^19^, ^20^ are less frequent in adult cases,^21^ and persistent cough is often the sole clinical manifestation.^22^ Consistent with previous reports, our study found few factors associated with atypical pathogen infections, including the presence of pneumonia and a close contact history. In contrast, the disease probability predicted by physicians was correlated with the detection rate of atypical pathogens in patients with a fever and cough, although the probabilities were occasionally overestimated. This study was unable to determine the cause of the overestimation, and an additional study will be required in order to confirm the current results and investigate the cause of overestimation.

Regarding the JRS diagnostic scoring criteria for atypical pathogen infections among adult pneumonia patients, Watanabe et al^23^ performed a prospective study with 1875 adult pneumonia cases and reported that the sensitivity and specificity were 79.2% and 63.7%, respectively, for the criteria without laboratory tests and 71.0% and 74.4%, respectively, for the criteria with laboratory tests. In contrast, Ishida et al^24^ performed a prospective study with 800 adult pneumonia cases and reported that the sensitivity and specificity were 77.0% and 93.0%, respectively, for the criteria with laboratory tests. That study's results showed a moderate sensitivity and high specificity when using criteria with laboratory tests for pneumonia patients. The current study showed that the sensitivity was higher with both criteria than in previous studies.

Recently, molecular identification technology has dramatically improved, and most bacteria and viruses can be automatically analyzed at acute care facilities by automated molecular identification systems within an hour after a few minutes of preparation. In the present study, we used two molecular rapid identification systems. The FilmArray® system and the FilmArray® Respiratory Panel tests are approved in many countries. FilmArray® can detect many respiratory pathogens with high sensitivity, and a variety of pathogens were detected in the present study. The GENECUBE system and GENECUBE Mycoplasma are approved in Japan and can detect *M pneumoniae*. The easy and rapid determination of the pathogen's information has made such knowledge available during the initial evaluations, along with other rapid testing results, and an improvement in the rate of appropriate antimicrobial use was noted in a previous observational study of pediatric pneumonia cases .^14^ During the study period, antimicrobial agents for atypical pathogens (macrolides, tetracyclines, or quinolones) were prescribed in 97.3% of *M pneumoniae*‐positive pneumonia cases (217/223) at the initial evaluation, while antimicrobial agents for atypical pathogens were prescribed only in 10.5% of 152 *M pneumoniae*‐negative pneumonia cases. The efficacy of rapid molecular identification was also proven by a randomized clinical trial for hospitalized adults with lower respiratory tract infection^25^, and its efficacy in reducing the duration of antimicrobial use (7.0 days vs 8.0 days, *P* < .001) and hospital stay (8.0 days vs 9.0 days, *P* < .001) was reported. Of note, we do not consider it appropriate to perform these molecular assays for all patients with a fever and cough because of the expense. These molecular examinations might be needed, especially for patients predicted as high for atypical pathogens infections by physicians, and immunocompromised patients.

Several limitations associated with the present study warrant mention. First, the current study was only conducted in two Japanese teaching hospitals across a period of approximately one and a half years. The epidemiology of atypical pathogens differs based on country,^26^ season,^27^, ^28^, ^29^ and year.^27^, ^30^ Therefore, the findings of the current study may not be generalizable to other settings. Second, we analyzed *M pneumoniae*, *B pertussis*, and *C pneumoniae* as atypical pathogens. While the detection of *M pneumoniae* was performed with two molecular assays, *B pertussis* and *C pneumoniae* were evaluated only through multiplex polymerase chain reaction assays. In addition, there were 43 patients (22.8%) with preceding antimicrobial use among atypical cases of pathogen‐negative respiratory tract infection. Therefore, the presence of false‐negative cases in this study cannot be ruled out.^31^ Finally, we did not analyze *Legionella pneumophila* or other atypical pathogens, including *B parapertussis*,^32^ *B holmesii*,^33^ and *C psittaci*,^34^ although they have been rarely identified in Japan.^35^, ^36^, ^37^

A comprehensive molecular analysis indicated that atypical pathogens were detected in only 10% of patients presenting with a fever and cough for more than 3 days. While physicians’ predictions were associated with the detection of atypical pathogens, overestimation was observed.

## CONFLICT OF INTERESTS

This study was supported by TOYOBO Co., Ltd. The funder provided fees for research expenses and GENECUBE assays. The funding source had no role in the design, practice, or analysis of this study. The authors have no conflicts of interest to disclose with respect to this research.
